# Bright tongue sign in patients with late-onset Pompe disease

**DOI:** 10.1007/s00415-019-09455-1

**Published:** 2019-06-29

**Authors:** Chafic Karam, Diana Dimitrova, Elizabeth Yutan, Nizar Chahin

**Affiliations:** 1 Department of Neurology, Oregon Health and Science University, 3181 SW Sam Jackson Park Rd., Portland, OR 97239 USA; 2 Department of Diagnostic Radiology, Oregon Health and Science University, Portland, OR USA

**Keywords:** Pompe, MRI, Tongue, Neuromuscular disorders

## Abstract

**Background:**

Late-onset Pompe disease (LOPD) is an often misdiagnosed inherited myopathy for which treatment exists. We noticed a bright tongue sign on brain MRIs of two patients who were admitted to the ICU for respiratory failure of unclear origin, and who were eventually diagnosed with LOPD. This led us to systematically review brain MRIs of patients with LOPD and various other neuromuscular disorders (NMD).

**Materials and methods:**

Chart and brain MRI review of patients with LOPD and other NMD.

**Results:**

Abnormalities of the tongue were observed in 11/33 of the patients studied. In 10/11 patients, no comments were made with regard to the tongue abnormalities in the radiology report. Bright tongue sign was seen in 4/6 patients with LOPD and 4/28 patients with other NMD. Tongue atrophy was seen in 3/6 patients with LOPD and 6/28 patients with other NMD.

**Conclusion:**

Tongue abnormalities on brain MRI are common in LOPD compared to other NMD. These abnormalities are not usually reported by the radiologist. Particular attention to the tongue when reviewing brain MRIs may be an important clue for diagnosis of a patient’s muscle weakness. A larger study is suggested to evaluate the sensitivity and specificity of tongue abnormalities in patients with LOPD.

## Introduction

Brain MRIs are commonly obtained in the neurology clinic for a variety of symptoms. The tongue is visible on the sagittal brain MRI and its appearance may give important clues on the patient diagnosis. Unfortunately, tongue abnormalities are usually missed or ignored by neurologists and radiologists when evaluating brain MRIs [1]. Furthermore, studies looking at the tongue on brain MRIs are scarce and in general limited to case reports and, occasionally, case series [2–6]. In the recent years, we have been consulted on two patients who were admitted to the intensive care unit (ICU) because of respiratory failure. Both patients were eventually diagnosed with late-onset Pompe disease (LOPD). The first patient was reported elsewhere and did not have a diagnosis at time of admission [1]. The other patient carried a misdiagnosis of facioscapulohumeral dystrophy (FSHD). In both these patients with LOPD, we noticed a striking bright tongue on T1 brain MRI. Those MRIs were initially requested by the primary team as part of workup for weakness. The tongue abnormalities were not noticed or were ignored by the radiologists and neurologists who initially evaluated the MRIs. Paying attention to these tongue abnormalities may have led to the correct diagnosis earlier. In this study, we aimed to systematically review the tongue appearance on brain MRIs in our patients with Pompe disease and other various neuromuscular disorders and study (1) how frequent tongue abnormalities are, (2) what type of tongue abnormalities are present, and (3) whether they were reported on original radiological or neurological interpretation.

## Methods

The Oregon Health and Science University Institutional Review Board (OHSU IRB #15998) approved this retrospective study. We reviewed the charts of patients seen at the OHSU ALS and Neuromuscular Disease Center between 7/1/2006 and 7/1/2016. We selected the patients who carried the diagnosis of LOPD, amyotrophic lateral sclerosis (ALS), primary lateral sclerosis (PLS), inclusion body myositis (IBM), myotonic dystrophy (MD), oculo-pharyngeal muscular dystrophy (OPMD), and FSHD. Those patients were specifically selected as they are relatively common and frequently complain of dysphagia. MRIs were ordered for various reasons such as headache, difficulty walking, etc. The data were obtained after query of electronic medical records. Only patients who carried one of the diagnosis cited above and had a sagittal T1 brain MRI that was available to us to review were selected. One of us (CK) verified the patients’ diagnosis by reviewing the history and clinical examination, available blood and genetic tests, and EMG findings. Patients who carried the diagnosis, but in whom we did not feel that the diagnosis was confirmed were not included. We also selected 36 patients who were age-controlled and who had brain MRIs for headache, altered mental status, chronic inflammatory demyelinating polyradiculoneuropathy (CIDP), acetylcholine receptor antibody-positive myasthenia gravis (MG), peripheral neuropathy, Lambert Eaton myasthenic syndrome (LEMS), POEMS, epilepsy and multiple sclerosis as a control group.

### Data collected

We specifically noted the age and gender of the patients, date of onset of symptoms, date of diagnosis, date of brain MRI, reason of brain MRI, official radiology read, whether there was dysarthria, dysphagia, neurological examination including tongue strength, whether the patient had limb vs bulbar onset in case of ALS, whether the patient was on enzyme replacement therapy in case of LOPD and whether EMG of the tongue was performed and the findings.

### MRI brain review

We reviewed the tongue appearance on T1-weighted MRI in the midsagittal brain. We looked specifically for abnormalities in the shape, size, position and internal structures of the tongue. Date, reason for the study and official radiology read was noted. One of us, a neuroradiologist (EY) reviewed the MRIs blinded of the actual diagnosis. We could not objectively evaluate the size or volume of the tongue because the MRIs were not specifically done to assess the tongue and not all of the tongue could be seen, hence the impression of tongue hyperintensity and atrophy on T1 images was done qualitatively. A tongue not touching the posterior palate was considered atrophic unless the mouth of the patient was open.

## Results

Between 7/1/2006 and 7/1/2016, 725 patients carried the diagnosis of either late-onset Pompe disease, ALS, PLS, IBM, MD1, OPMD, and FSHD. After reviewing the charts, 35 patients who had sagittal brain T1-weighted MRI which was available for us to review were selected. The rest of the patients were excluded from the study either because they did not have a brain MRI available for review, the brain MRI did not have a sagittal T1-weighted image, or we felt that their diagnosis was not correct. Of these 35 patients, 6 had Pompe disease, 9 had bulbar-onset ALS, 8 had limb onset ALS, 3 had PLS (1 with bulbar onset), 1 had FSHD, 4 had IBM and 4 with MD1. Patients’ characteristics with MRI findings are summarized in the Table 1. In two patients, the tongue could not be interpreted because of either severe motion artifact or because the tongue was cut off the view.

**Table 1:** Patient findings

	Diagnosis	Gender	Dysarthria	Dysphagia	Tongue weak	Bright tongue	Tongue atrophy	EMG tongue
p1	Pompe	M	No	No	No	Yes	No	No
p2	Pompe	M	No	No	No	Yes	Yes	No
p3	Pompe	F	No	Yes	Yes	Yes	No	No
p4	Pompe	F	No	No	No	No	No	No
p5	Pompe	M	No	No	No	No	No	No
p6	Pompe	M	No	No	No	No	No	No
p8	ALS (B)	M	Yes	Yes	Yes	Yes	Yes, mild	Yes, abnormal
p9	ALS (B)	F	Yes	Yes	Yes	No	No	No
p10	ALS (B)	F	Yes	Yes	Yes	No	No	Yes, abnormal
p11	ALS (B)	M	No	Yes	Yes	No	Yes, mild	Yes, abnormal
p12	ALS (B)	F	Yes	Yes	Yes	No	No	No
p13	ALS (B)	F	Yes	Yes	Yes	No	No	No
p14	ALS (B)	F	No	Yes	Yes	No	Yes	No
p15	ALS (B)	F	Yes	Yes	Yes	Yes	Yes	Yes, abnormal
p16	ALS (L)	M	Yes	Yes	Yes	No	No	Yes, normal
p17	ALS (L)	M	Yes	Yes	Yes	No	No	No
p18	ALS (L)	F	Yes	Yes	Yes	Yes	No	No
p19	ALS (L)	F	No	No	No	No	No	Yes, normal
p20	ALS (L)	F	No	Yes	No	No	No	No
p21	ALS (L)	M	No	No	No	No	No	No
p22	ALS (L)	M	No	No	No	No	No	No
p23	ALS (B)	F	Yes	Yes	Yes	Artifact	Artifact	Yes, abnormal
p24	ALS (L)	F	No	No	No	No	No	Yes, normal
p25	PLS (L)	M	Yes	Yes	No	No	No	Yes, normal
p26	PLS (L)	F	Yes	Yes	No	No	No	Yes, normal
p27	PLS (B)	F	Yes	Yes	No	No	No	Yes, normal
p28	FSHD	F	No	Yes	No	No	No	No
p29	IBM	F	No	Yes	No	No	No	No
p30	IBM	M	No	Yes	No	No	No	No
p31	IBM	F	No	No	No	No	No	No
p32	IBM	M	No	No	No	No	No	No
p33	MD1	M	No	No	No	No	No	No
p34	MD1	F	No	Yes	Yes	NS	NS	No
p35	MD1	F	No	No	No	Yes	Yes	No
p36	MD1	M	No	No	No	No	No	No

*P* patient, *M* male, *F* female, *ALS* amyotrophic lateral sclerosis, *PLS* progressive lateral sclerosis, *IBM* inclusion body myositis, *MD1* myotonic dystrophy type 1

### MRI findings

**Fig. 1:**
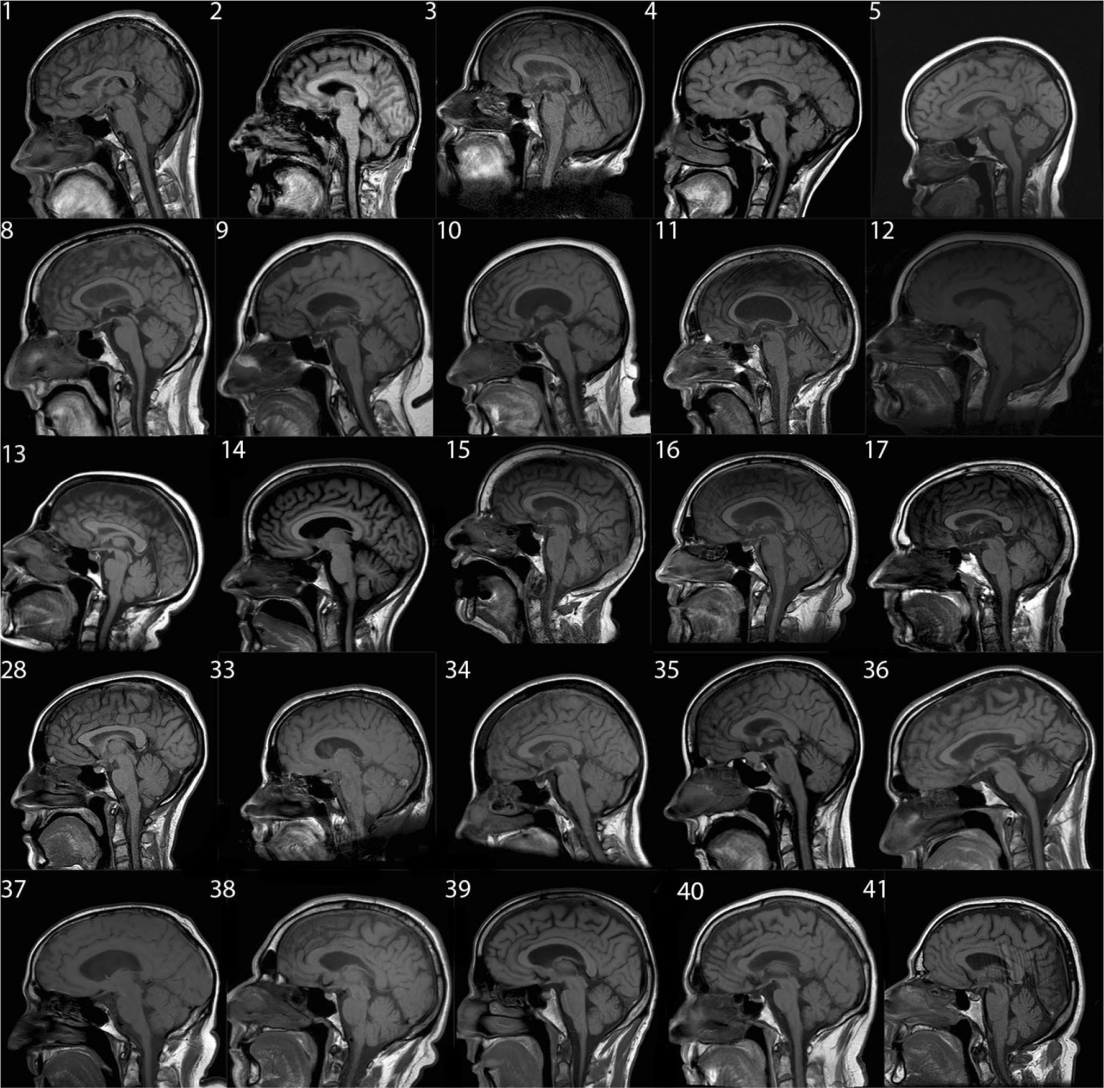
Representative sagittal brain T1 MRIs. Patients’ diagnosis, description and tongue findings are in the table. Patients 37 through 41 are normal controls

Patient had brain MRIs for reasons either directly related to their disease or related to something else such as headache or to rule out stroke. All patients underwent 1.5 T brain MRIs. Abnormalities of the tongue were observed in 11/33 of the patients. However, in only one patient the tongue was reported as abnormal in the radiology report. Representative MRI cases are shown in the Fig. 1.

#### Late-onset Pompe patients

All patients had MRI after onset of symptoms and diagnosis. Four of the six Pompe patients had abnormal tongue signal and 2/6 had tongue atrophy. However, only one of those patients had dysphagia and none had dysarthria. Length of disease did not seem to play a role in this small cohort. Tongue signal abnormalities or “bright tongue” was most striking in Pompe patients in comparison with the rest of the patients studied.

#### ALS patients

All patients had MRI after onset of symptoms. Nine patients had bulbar onset. In one patient, the tongue appearance could not be interpreted because of severe artifact. Two of eight patients with bulbar ALS patients had abnormal tongue signal and 4/8 had tongue atrophy. When performed, tongue EMG was reported as abnormal in all patients. In the limb-onset ALS, one of eight patients had tongue abnormalities (mild T1 hyperintensity) on brain MRI. That patient had bulbar symptoms at time of MRI. Tongue atrophy was most pronounced in ALS patients in comparison with the rest of the patient studied. The tongue atrophy resulted in an angulated shape instead of the curvilinear appearance of the tongue, which is seen in normal patients.

#### PLS patients

None of the three PLS patients had tongue abnormalities on MRI. One of those patients had bulbar-onset PLS. None of those patients had abnormal tongue EMG.

#### FSHD patient

Only one patient with FSHD in our cohort had brain MRI with sagittal T1 imaging. The patient had dysphagia but no dysarthria. There tongue did not fully occupy the oral cavity but was not significantly atrophic. There was no signal abnormality.

#### IBM patients

None of the four patients with IBM had tongue abnormalities on brain MRI. Two patients did have dysphagia.

#### DM patients

All DM patients were of type 1. One of the patients had bright tongue and atrophy. Another patient had bright tongue only. The third patient had severe motion artifact and the findings could not be interpreted. In the fourth patient, the MRI was cut and the tongue could not be apparent.

#### OPMD

None of our OPMD patients had brain MRI available for us to review.

#### Control patients

None of the control patients had tongue signal abnormalities or atrophy. In one patient, the tongue did not touch the posterior palate but the patient has his mouth open during the MRI study.

## Discussion

This study shows that the tongue can be easily seen on routine brain MRI and tongue abnormalities are more common in Pompe disease than other MD. These abnormalities are frequently ignored by radiologist and neurologists, since usually the tongue appearance is not of interest in brain MRIs. T1-weighted sequences are useful when evaluating the muscles because sensitive enough to detect both muscle atrophy and increased signal related to fatty content, and because they are usually obtained routinely. Normally, the tongue has an elliptical shape, occupies the entire oral cavity touching the posterior palate and has two curvilinear bands parallel to the mucosal surface [2].

In our study, most patients with Pompe disease had a bright tongue sign. This can be a clue to the correct diagnosis, especially when patients are admitted to the ICU for respiratory failure of unclear origin. Furthermore, patients frequently undergo brain MRI for evaluation of weakness and the clinician paying attention to tongue abnormalities could get important clues to the underlying disease.

In a study on whole-body muscle MRI in 20 patients with LOPD, tongue abnormalities were reported in most patients although the exact number was not specified [4]. In the study, the authors commented that “muscle changes seen in T1-weighted sequences consisted essentially of fat replacement which manifests as bright signal, but without severe retraction of the muscle corpus (i.e. muscle shape and volume were preserved although the inner content changed)”. Our study shows that not all patients with LOPD had tongue involvement and that tongue atrophy was present in some of the patients. We do not have a good explanation of why there is this discrepancy between the two studies. However, the other authors have reported on one patient with moderate lingual weakness and diffuse fatty infiltration of the tongue [3]. In contrast to patients with bulbar ALS, patients with Pompe and abnormal tongue findings on brain MRI did not have dysarthria. Only two out of the seven patients with Pompe complained of dysphagia. One of those had tongue abnormalities on brain MRI.

To our knowledge, tongue abnormalities on brain MRI in MD patients have not been described. But our findings suggest that tongue abnormalities are probably common in patients with MD type 1. One study evaluated the tongue in 151 FSHD patients and found tongue atrophy and signal abnormalities on brain MRI in 7 patients [6]. Only 1 of our 45 FSHD had a brain MRI and we cannot comment further on this finding. Abnormalities of tongue on brain MRI in ALS patients have been studied [2]. The authors found tongue atrophy in 14 of 16 ALS patients and increased signal intensity in 12 of 16 ALS patients. This is in contrast to our patient population where we found tongue abnormalities in only 5/16 patients. This may be explained by the fact that our patients had brain MRIs earlier in the course of their disease.

Tongue abnormalities on brain MRI are not specific to LOPD and can be seen in other disorders that affect the tongue such as ALS and MD1. Hence, we do not think that analyses of the tongue on imaging will help differentiate between various neuromuscular disorders. However, tongue abnormalities on brain MRI, when evaluating patients for a gait disorder for example, can help orient the physician to the correct diagnosis.

Major limitations of this study are the retrospective nature with a minority of the patient having a brain MRI available for us to review and the lack of objective measure of the tongue size. The small number of patients makes the calculation of frequency, sensitivity and specificity inadequate. The lack of dedicated tongue MRI makes objective measurement of the tongue impossible.

The purpose of this study is not to suggest that patients with neuromuscular disorders should undergo brain MRI to assess for tongue abnormalities and in our practice, we do not routinely order brain MRI for evaluation of a neuromuscular patient. However tongue abnormalities found on brain MRI ordered for other reasons can give clues for the diagnosis of a neuromuscular disorder.
